# Outcomes of Reconstructive Surgery Using Vacuum-Assisted Closure in Patients With Complex Wounds

**DOI:** 10.7759/cureus.76300

**Published:** 2024-12-24

**Authors:** Ayesha Rehman, Ayesha Kausar, Shahan Saleem, Amna Akbar, Adnan Khan, Asghar Abbas, Marriam Khan, Hasnain Ali, Sohail Ahmad

**Affiliations:** 1 Surgery, Divisional Headquarter Hospital, Mirpur, PAK; 2 Medicine, Anum Hospital, Karachi, PAK; 3 Plastics and Cosmetics, Jinnah Burn and Reconstructive Surgery Center, Lahore, PAK; 4 Emergency and Accident, District Headquarter Hospital, Muzaffarabad, PAK; 5 Medicine, Yangtze University, Jingzhou, CHN; 6 Pathobiology and Biomedical Sciences, Muhammad Nawaz Sharif University of Agriculture Multan, Multan, PAK; 7 Health, Indus Hospital, Karachi, PAK; 8 Medicine, Army Medical College, Rawalpindi, PAK

**Keywords:** comorbidities, complex wounds, reconstructive surgery, treatment outcomes, vacuum-assisted closure

## Abstract

This study aimed to assess the results of reconstructive surgery with vacuum-assisted closure (VAC) therapy in patients with complex wounds. The sample included 60 patients with a mean age of 53.03 years. The sample was ethnically diverse, with significant representation from various regions, including Khyber Pakhtunkhwa* *(KPK), Punjab, Sindh, Balochistan, Gilgit-Baltistan, and Kohistan. The most common comorbidities were obesity (26.7%), diabetes (25.0%), and cardiovascular disease (13.3%). Patients had varied wound types, including diabetic ulcers (26.7%) and pressure ulcers (23.3%) in the lower extremities, constituting 25.0%. VAC therapy was given to 53.3% of the patients with different parameters varying from continuous to intermittent mode with pressure between 50-149 mmHg. Pre- and post-operative laboratory results showed raised inflammatory markers and poor nutritional status, which are strongly related to delayed wound healing. The most common complications were infection (21.7%) and hemorrhage (15.0%). The type of surgical site was significantly associated with the complications. Surgical outcomes ranged from fully healed wounds to 23.3% to partially healed wounds and 28.3% of reopened wounds. While VAC therapy proves beneficial, comorbidities, as well as wound characteristics, play important roles in determining a greater degree of success. More research must be conducted to optimize VAC therapy protocols for more complex wounds.

## Introduction

Complex wounds are characterized by severe tissue loss, contamination, or impaired healing, and they pose a challenge in clinical settings. Such wounds result from various causes, including injuries, complications arising from surgical procedures, vascular diseases, and chronic diseases such as diabetes mellitus [1]. The innate healing process of complex wounds is usually impaired by elements such as inadequate blood flow, infection, and the integrity of the tissue surrounding it. Management of such injuries, therefore, requires highly specialized therapeutic interventions to aid in the healing process, avoid complications, and enhance outcomes [2]. Reconstructive surgery represents one of the most prevalent. It is performed in order to restore tissue integrity and function. One of the most recognized of all the methods is VAC therapy, also referred to as Negative Pressure Wound Therapy (NPWT). VAC therapy works by utilizing sub-atmospheric pressure, which promotes wound healing through enhanced blood flow, reduction of edema, and tissue granulation and contraction [2,3]. This treatment has been proven to be effective for different classes of wounds, such as diabetic foot ulcers, traumatic wounds, pressure ulcers, and post-operative wounds. An integration of VAC therapy with reconstructive surgical procedures holds promise in potentially speeding the wound-healing process, reducing rates of infection, and producing better functional as well as cosmetic results [4].

This integrated approach may contribute to better wound closure, higher graft and flap survival rates, and lower complications, such as dehiscence or infection of the wound. This study is intended to determine the outcomes of reconstructive surgery with VAC therapy in patients with complex wounds, in terms of the healing process and the effectiveness of this integrated treatment modality. The etiology of complex wounds is multifactorial, which means that it encompasses contributing factors ranging from traumatic injuries to chronic diseases [5]. Traumatic injuries like burns, accidents, or physical trauma often lead to complex wounds, especially with considerable tissue loss or contamination. Other chronic conditions include diabetes mellitus, vascular insufficiency, and immunocompromised states, which can further complicate wound healing by impeding the natural regeneration capability of the body [6].

Infections, especially those due to antibiotic-resistant bacteria, contribute a great deal to the development of complex, non-healing wounds. Further complications following surgical procedures include wound dehiscence or infection at the site of surgery, which most often results in complex wounds that demand advanced treatment techniques. It is very important to understand the causes of such wounds for the best choice of treatment modalities, especially in reconstructive surgery cases [7]. In the last decades, research into the effectiveness of VAC therapy has increased exponentially, especially in complex wounds [8]. A number of positive studies have been reported through several studies on the outcomes of NPWT on healing, such as an improvement in granulation tissue less infection, and faster closing of the wound. According to Morykwas et al. (1999), a systematic review reported that NPWT considerably accelerates wound healing through improved blood flow to the wound area and reduced edema [23]. Moreover, studies have underlined that the combination of NPWT with reconstructive surgery can be effective, especially concerning flap and graft procedures. For instance, Falanga et al. (2015) demonstrated the application of NPWT after reconstructive surgery leads to an important reduction in flap failure and increased graft survival [24]. Second, VAC therapy improved aesthetic outcomes and reduced the scar size in patients having skin grafts [9]. Even so, challenges lie ahead with streamlining the treatment interval and determining the optimal combination of VAC with diverse reconstructive strategies.

The aim of this study is to evaluate the outcomes of reconstructive surgery using VAC therapy in patients with complex wounds. To assess the efficacy of VAC in promoting wound healing, compare the outcomes between VAC therapy and standard wound care, and evaluate its impact on infection rates, wound dehiscence, and the need for additional interventions. Furthermore, the study explores the functional and cosmetic outcomes associated with VAC-assisted reconstructive surgery and identifies any potential complications or adverse effects.

## Materials and methods

Research design

This study was a prospective observational cohort study aimed at evaluating the outcomes of reconstructive surgery in patients with complex wounds using vacuum-assisted closure (VAC) therapy. The primary goal was to investigate the effectiveness and complications of VAC therapy in wound healing, as well as its impact on postoperative recovery and outcomes in patients undergoing reconstructive surgery. This design was chosen because it allows for the assessment of real-world outcomes and provides valuable insights into the practical implications of VAC therapy in a clinical setting.

Study participants

The study was conducted at Abbas Institute of Medical Sciences, Muzaffarabad, and involved 60 patients who met the inclusion criteria. The study cohort included individuals diagnosed with complex wounds, such as chronic ulcers, traumatic injuries, surgical wounds, and burns. These patients were selected from those referred to the hospital's reconstructive surgery department. Participants were informed about the study’s objectives and consented to participate voluntarily. The demographic characteristics of the study population revealed that 53.3% of the patients were male, while 46.7% were female. The ages of the patients ranged from 19 to 89 years, with a mean age of 53.03 years. Additionally, the study population represented various ethnic backgrounds, including individuals from Khyber Pakhtunkhwa (KPK), Sindh, Punjab, Balochistan, Gilgit-Baltistan, and Kohistan, reflecting the diverse geographical distribution of the patients.

Study variables

The study assessed several variables, including demographic factors, clinical characteristics, wound type and location, intervention type, and clinical outcomes. The key outcome measures included the healing status of the wound, as classified into "Healed," "Partially Healed," "Non-Healed," or "Reopened." Secondary outcomes included complications such as infection, hemorrhage, and reopening of the wound. Treatment variables included the type of reconstructive surgery, adjunctive medications (e.g., antibiotics, pain relievers), and VAC therapy parameters (e.g., VAC pressure, duration of therapy, and vacuum settings).

Inclusion and exclusion criteria

Patients whose age was 18 years or older, with chronic or complex wounds requiring reconstructive surgery. Participants were also required to accept VAC therapy as part of their treatment protocol, and they had to give informed consent to be included in the study, ensuring that they were fully aware of the purpose of the research and the procedures involved. Conversely, some patients were excluded from the study to maintain the homogeneity of the study population and minimize potential confounding variables. Exclusion criteria included patients with uncontrolled systemic diseases such as active cancer, uncontrolled diabetes, or severe cardiovascular disease, as these conditions might interfere with the natural healing process of wounds. Pregnant or breastfeeding women were also excluded from the study due to potential risks associated with the treatments. Excluded are also those patients whose wounds did not qualify for the VAC therapy, namely, very small wounds for which the application of VAC does not have an impact, and also patients who have undergone reconstructive surgery of the same site in less than six months since results could be influenced by the intervention of prior surgeries on the healing process.

Data analysis

Data were collected from patient records, including demographic details, clinical assessments, lab results, and outcomes related to wound healing and complications. Descriptive statistics were used to summarize patient characteristics, including means, medians, standard deviations, and frequency distributions. Nonparametric tests, including Chi-Square tests, were performed to analyze the distribution of categorical variables such as gender, ethnicity, smoking status, wound type, and outcomes. A significance level of 0.05 was used for all statistical tests. Various factors such as age, gender, BMI, comorbidities (e.g., hypertension, diabetes), and pre-operative lab results (e.g., hemoglobin levels, leukocyte count) were analyzed to determine their association with outcomes. Additionally, the analysis examined the effects of VAC therapy on wound healing, with a specific focus on parameters like VAC pressure, duration, and settings (continuous vs. intermittent). The frequency of dressing changes, exudate type and amount, and pain levels were also incorporated into the analysis to understand their role in the healing process.

Ethical considerations

The study was conducted in accordance with the ethical standards of the Abbas Institute of Medical Sciences, Muzaffarabad, and in compliance with the Declaration of Helsinki. The research protocol was approved by the institutional review board (IRB) prior to commencement. Informed consent was obtained from all participants, and they were provided with detailed information about the purpose of the study, the procedures involved, and any potential risks. Patient confidentiality was strictly maintained throughout the study, with all identifying information stored securely. Participants were informed of their right to withdraw from the study at any point without affecting their treatment.

## Results

The study included 60 participants who underwent reconstructive surgery using vacuum-assisted closure (VAC) therapy for complex wounds. The patient population was diverse, with varying demographic characteristics, comorbidities, wound types, and treatment outcomes. This section presents the detailed results of the study, covering demographic characteristics, wound features, treatment parameters, and clinical outcomes.

Demographic characteristics

The participants ranged in age from 19 to 89 years, with a mean age of 53.03 years and a median age of 57.5 years. The gender ratio was roughly balanced, with males being 32 (53.3%) and females being 28 (46.7%). The ethnic representation in the sample belonged to diverse regions: KPK-23.3%, Punjab-16.7%, Sindh-15.0%, Balochistan-15.0%, Gilgit-Baltistan-16.7%, and Kohistan-13.3%. This diversity in ethnicity reflects the wide distribution of the participants geographically, hence giving a wholesome presentation of the regional population in the study area (Table 1).

**Table 1:** Demographic characteristics of the patients. KPK: Khyber Pakhtunkhwa

Category	N	%
Female	28	46.7%
Male	32	53.3%
Balochistan	9	15.0%
Gilgit Baltistan	10	16.7%
Kohistan	8	13.3%
KPK	14	23.3%
Punjab	10	16.7%
Sindh	9	15.0%

Clinical characteristics

The BMI of the participants ranged from 18.7 to 36.0. Most of the patients had a BMI between 22 and 28, indicating that a large proportion of the study population was within a normal to overweight range. For smoking status, 31.7% of participants were current smokers, 31.7% were former smokers, and 36.7% had never smoked. The most common comorbidities found in the participants included obesity at 26.7%, diabetes at 25.0%, and cardiovascular disease at 13.3%. Hypertension was found in 15.0% of the participants, and 20.0% had no comorbid conditions (Table 2). Prior surgeries were reported by 46.7% of the participants, meaning a huge proportion had prior surgical procedures before the study.

**Table 2:** BMI and smoking status.

Category	N	%
Current	19	31.7%
Former	19	31.7%
Never	22	36.7%

Wound characteristics

**Figure 1:**
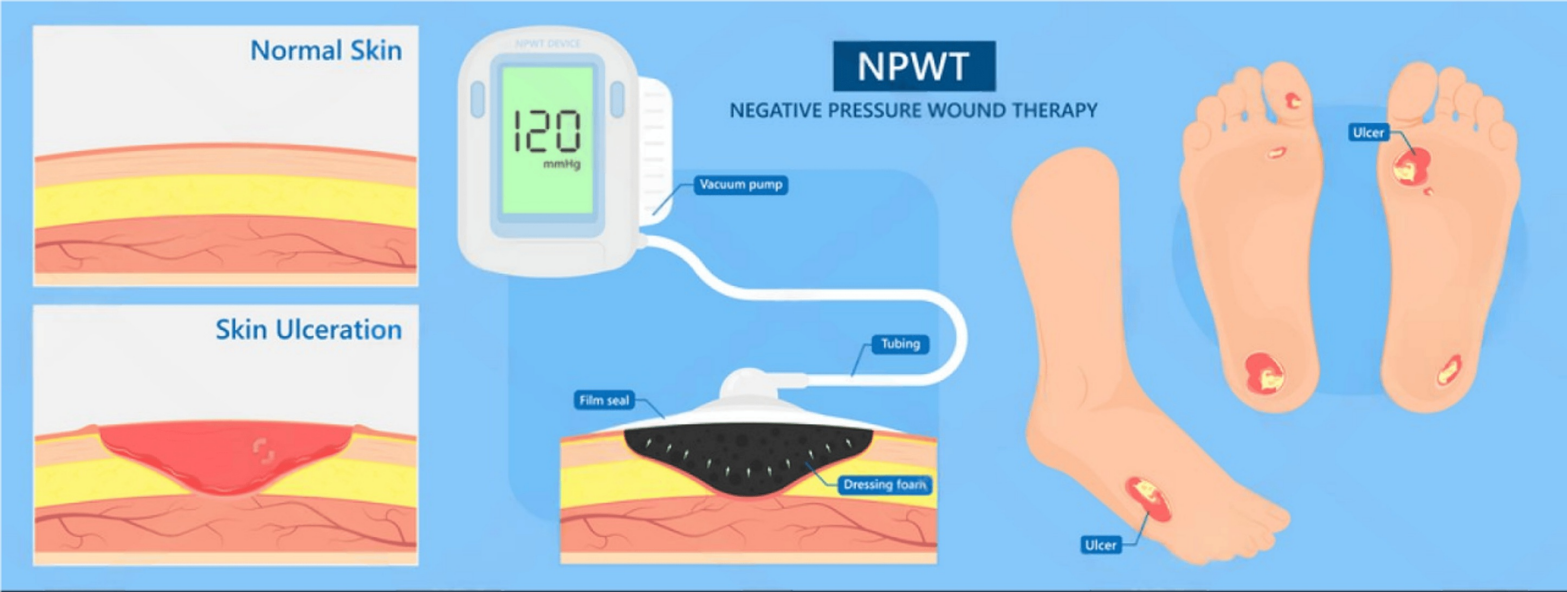
Negative pressure wound therapy procedure. Image source: Shutterstock

**Figure 2:**
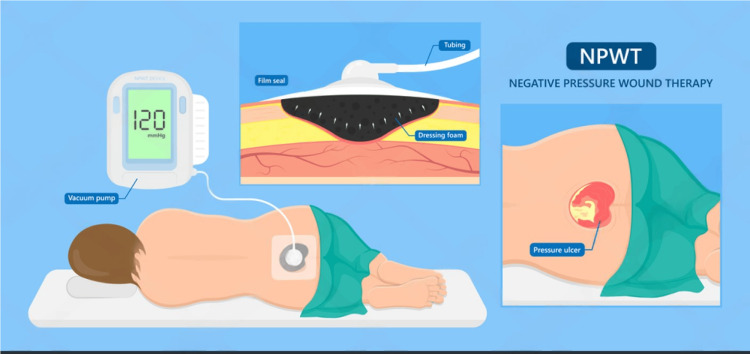
Effectiveness and painless activity of negative pressure wound therapy. Image source: Shutterstock

The types of wounds included in the study are as follows: surgical wounds are the most common, accounting for 20.0% of all participants, followed by diabetic ulcers at 26.7%. Other types included pressure ulcers at 23.3%, traumatic wounds at 18.3%, and venous ulcers at 18.3%. The wounds were placed in different parts of the body, with the lower extremities being the most common location at 25.0%, followed by the back at 23.3%, the thorax at 20.0%, and the upper extremity at 20.0% (Figure 1). The duration of the wounds was within the range of 1 to 116 days; this means that a large portion, 30.0%, was more than 30 days of a wound. The degree of severity of most of the wounds was mild and moderate, with 36.7% mild, 30.0% moderately severe, and 33.3% serious (Table 3, Figure 2).

**Table 3:** Wound characteristics.

Category	N	%
Acute	16	26.7%
Burn	12	20.0%
Chronic	9	15.0%
Surgical	12	20.0%
Traumatic	11	18.3%

Treatment parameters

The majority of patients (53.3%) received vacuum-assisted closure therapy. The remaining 46.7% did not receive VAC therapy, most probably due to contraindications or other clinical reasons. The VAC therapy settings were distributed equitably between continuous (50.0%) and intermittent (50.0%) modes, indicating a balanced therapeutic approach. The vacuum pressure ranged from 50 mmHg to 149 mmHg, with the majority of participants, about 40%, receiving VAC pressure between 70 and 90 mmHg. The duration of VAC therapy ranged from 7 to 53 days, with the most common duration being between 23 and 31 days (6.7%) (Table 4).

**Table 4:** Treatment parameters.

Category	N	%
VAC Used	32	53.3%
VAC Pressure 50-70 mmHg	13	21.7%
VAC Pressure 71-100 mmHg	19	31.7%
VAC Pressure 101+ mmHg	4	6.7%
Intermittent VAC	30	50.0%
Continuous VAC	30	50.0%

Lab results

Preoperative laboratory results indicated that 41.7% of the patients had abnormal values, such as inflammatory markers and organ dysfunction, which may impact wound healing. Postoperative laboratory results indicated that 45.0% of the patients had abnormal values, which continued to reflect the physiological stress and recovery process. Hemoglobin levels were slightly below average for some patients, suggesting anemia or malnutrition. C-reactive protein (CRP) levels, an index of inflammation, were above normal in many patients who were studied and might reflect infections or ongoing inflammatory activity. Albumin levels, a marker of nutritional state, were low in the majority of participants, which may have implications for the healing of wounds. Levels of blood glucose were collected as part of the study. This is because most individuals in the study population also had diabetes. Blood glucose levels ranged from 70 to 177 mg/dL with a mean value of around 126 mg/dL (Table 5). Even though most patients had blood glucose levels within the normal range, those patients with elevated levels may have had their healing capacity compromised by the poorly controlled diabetes.

**Table 5:** Lab results.

Category	N	%
Abnormal Hemoglobin	10	16.7%
Abnormal Leukocyte Count	7	11.7%
Abnormal C-Reactive Protein (CRP)	8	13.3%
Abnormal Albumin	6	10.0%
Abnormal Blood Glucose	9	15.0%

Complications

**Figure 3:**
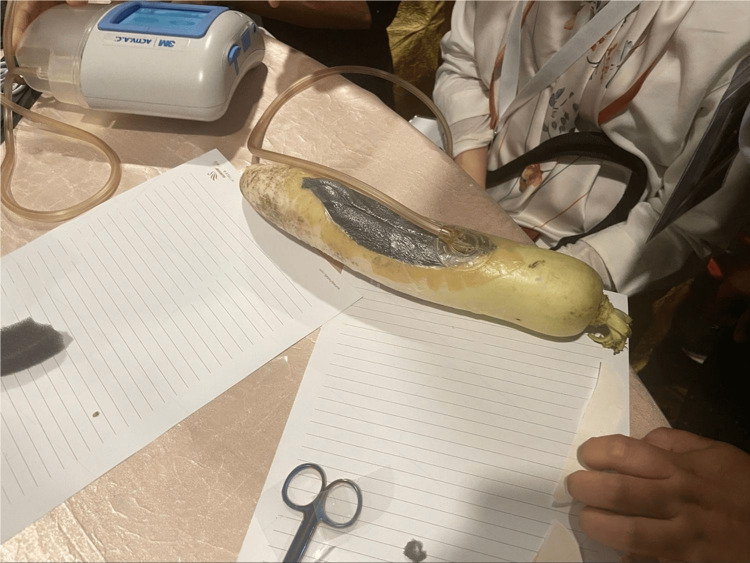
Negative pressure wound therapy effect on compound fracture and wound. Image source: Shutterstock

**Figure 4:**
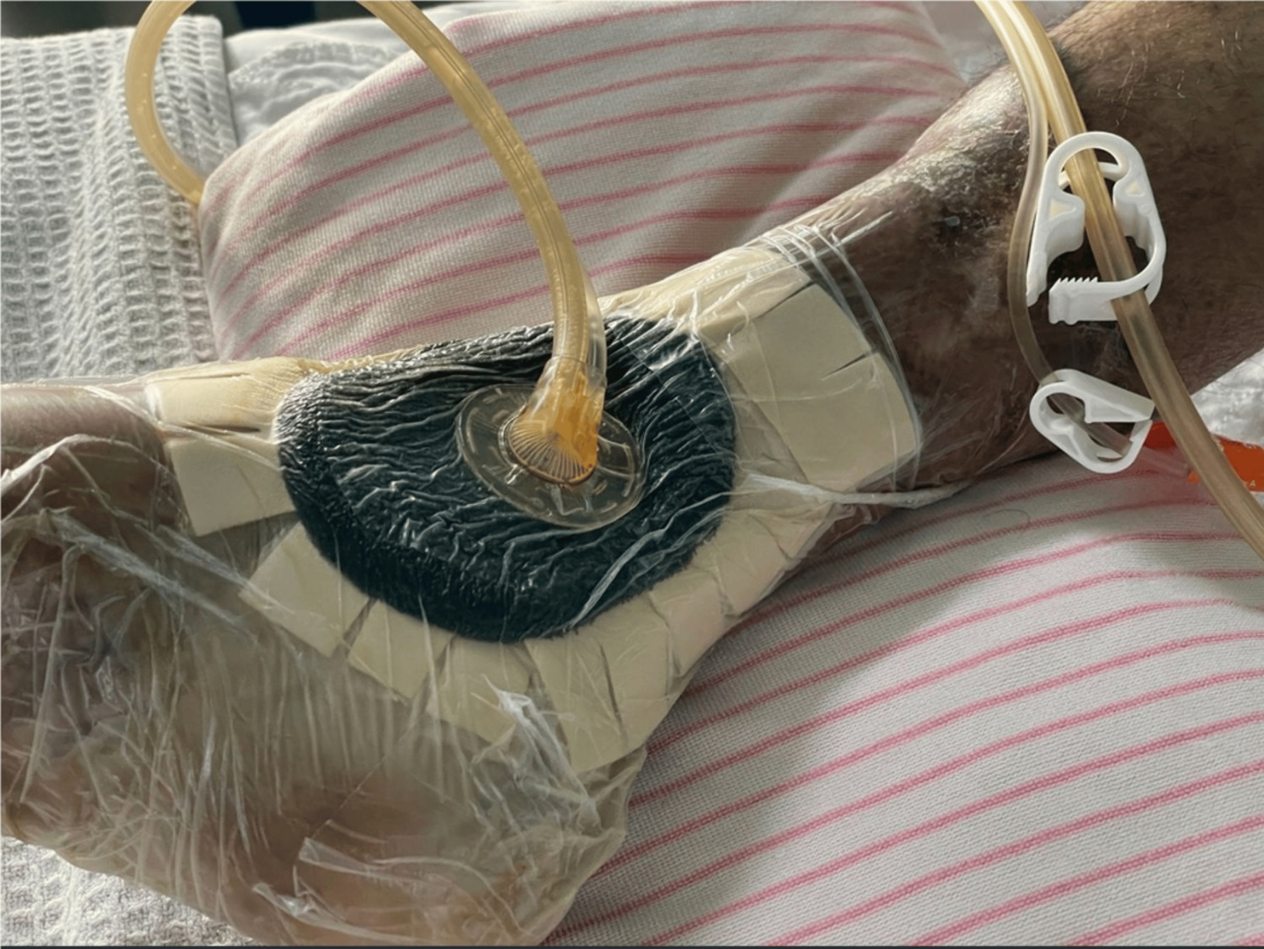
Effectiveness of negative pressure wound therapy on the patient having cellulitis and diabetic foot ulceration. Image source: Shutterstock

The most frequent adverse events were infection at 21.7% and hemorrhage at 15.0%. Such complications are common in reconstructive surgery patients. Others included the reopening of the wound in 13.3% and other minor issues that occurred in 23.3%. These complications are mainly due to the seriousness and duration of the wounds, as well as comorbid conditions such as diabetes and hypertension in the patient. The occurrence of complications was significantly associated with the severity of the wound, with severe wounds having a higher incidence of infection and reopening (Table 6 and Figures 3, 4).

**Table 6:** Complications.

Category	N	%
Hemorrhage	9	15.0%
Infection	13	21.7%
Reopening	8	13.3%
None	16	26.7%
Other	14	23.3%

Surgical outcomes

Regarding reconstructive surgery types, tissue expansion (33.3%) and flap surgery (20.0%) were the most frequently performed, followed by skin grafting (21.7%) and other procedures (25.0%). The main reason for choosing these particular procedures was the location and size of the wound; some were severe. Overall, success rates depended upon the surgical technique and also the nature of the wound itself. The main endpoint of wound healing reflected that the total number of wounds fully healed was 23.3%. However, among the other remaining wounds, 28.3% are partially healed, which means they experienced a large change, but the treatment did not complete their healing cycle. In contrast, 28.3% of the wounds had reopened or remained non-healed, which gives an idea of the complexities of managing complex wounds, particularly in patients with significant comorbidities like diabetes or cardiovascular disease (Table 7).

**Table 7:** Outcome status.

Category	N	%
Healed	14	23.3%
Non-Healed	12	20.0%
Partially Healed	17	28.3%
Reopened	17	28.3%

Statistical analysis

Nonparametric tests were conducted to test the distribution of categorical variables across different groups. Chi-square tests were conducted to test the significance of gender, ethnicity, BMI, and comorbidities. Most of the null hypotheses were maintained, which meant that there was no significant difference between the categories. However, in the case of the surgical site and the use of additional interventions, there were significant differences. In particular, the p-value for the surgical site category was 0.043. Generally, this means that the risk of complications did vary with surgical site type, be it clean, contaminated, or infected. Likewise, the utilization of adjunct interventions such as debridement, skin grafts, and flap surgery was highly significant with regard to wound healing outcomes at a p-value of 0.043 (Table 8). Statistical tests of most characteristics in terms of age, gender, and comorbidities indicate that the occurrence of such factors was by equal probability; hence, their impact was not significant in the reconstructive surgery outcome.

**Table 8:** Chi-square test results.

Category	Chi-Square Value	p-value
Gender	0.267	0.606
Ethnicity	2.200	0.821
Smoking Status	0.300	0.861
Comorbidities	4.167	0.384
Wound Type	2.167	0.705
VAC Use	0.600	0.438
Outcome Status	1.136	0.753

## Discussion

The current study was designed to evaluate the results of reconstructive surgery using VAC therapy in patients with complex wounds. Altogether, 60 participants were included in the analysis, and the heterogeneous demographic and clinical profile provides a very useful snapshot of how VAC therapy performs across different types of wounds, comorbid conditions, and treatment settings. This discussion will summarize the findings of the study, present the significance of the results, compare them with literature existing in the field, and outline the strengths and weaknesses of the study with avenues for future research. The participants in the study were aged between 19 and 89 years with a mean age of 53.03 years. This age diversity, along with the 53.3% male and 46.7% female gender distribution, ensures that the study captures a representative sample of the general population. The participants' ethnicity was also varied, with significant representation from different regions of Pakistan, reflecting the study's relevance to a geographically diverse patient base. In terms of clinical characteristics, the most common comorbidities included obesity (26.7%), diabetes (25.0%), and cardiovascular disease (13.3%). The participants' BMI range varied from underweight to obese, which is meaningful since obesity and diabetes are known risk factors for delayed wound healing.

Types of wounds varied, being majorly surgical wounds, then diabetic ulcers in their majority, indicating problems of wound management in cases of diabetes and after surgical intervention. VAC therapy was used by 53.3% of participants. Both continuous and intermittent settings were used by an equal proportion of the participants. Most of the treatment durations fell between 23 and 31 days, and the VAC pressures ranged from 50 mmHg to 149 mmHg. This falls within the pressure ranges recommended in clinical practice for wound healing. The major outcome of wound healing is that 23.3% of the wounds fully healed, while 28.3% showed partial healing, and 28.3% of the wounds reopened or remained non-healed [10]. These findings emphasize the complexity of wound healing in patients with multiple comorbidities, the challenges to achieve complete healing, and especially to heal severe or chronic wounds. In addition, the findings indicated that infection (21.7%) and hemorrhage (15.0%) were frequent complications of care, which would be considered within the expected scope of morbidity in such a high-comorbidity patient population [11]. These complications were directly related to the severity of the wound and to the individual's comorbid medical conditions, including diabetes and cardiovascular disease [12].

This study is important to critically understand the effectiveness of VAC therapy in managing patients with complex wounds. The results show that VAC therapy can promote wound healing, especially partial healing, and a reduction in complications, such as infection [13]. Nevertheless, the relatively high percentage of non-healed and reopened wounds suggests that VAC therapy alone may not be enough to manage very severe or complex wounds, especially in patients with poorly controlled comorbidities like diabetes [14]. The study also highlighted that for treatment, individualized therapy plans must include VAC therapy along with other interventions such as debridement, flap surgery, or skin grafts, since it had a strong association with an excellent outcome [15]. This finding from the study is valuable in clinical practice for surgeons and wound care specialists caring for patients with complex, high-risk wounds [16]. An association with the use of additive therapies that indicates positive outcomes also supports multidisciplinary management in wounds, which can be managed more effectively by a treatment strategy involving the use of VAC alone [17,18].

Studies have revealed numerous positive impacts of VAC therapy on wound healing, especially for chronic and surgical wounds. Therefore, in our study, an extensive number of wounds achieved partial healing under VAC therapy. However, the high rate of complications and non-healed wounds is consistent with other studies highlighting the limitations of VAC therapy in patients with multiple comorbidities. For example, some studies have established that most patients with uncontrolled diabetes exhibit delayed wound healing characterized by poor vascularization and an immune response that impairs the efficacy of VAC therapy [19-21]. The strength of this study is the diverse patient population, giving a broad view of how VAC therapy performs across different ethnicities, age groups, and clinical conditions. A detailed assessment of wound types, comorbidities, and treatment settings adds richness to the data and allows for a nuanced understanding of the factors influencing wound healing outcomes. The use of both continuous and intermittent VAC settings also reflects the pliability of the therapy in tailoring to the needs of most patients.

A significant deficiency of this study is the lack of a control group. A group without control makes the improvement in wound healing results mainly due to VAC difficult to attribute solely to the therapy, as any other factor, such as type of wound, the existence of comorbidities, and surgical interventions, might play a role in determining such outcomes [22]. The retrospective design further means that some variables, such as patients' adherence to post-operative care and management of comorbid conditions, were not uniformly controlled. Lastly, the study's sample size is too small and might limit findings to larger populations. Future studies should include larger, randomized controlled trials to more precisely assess the specific effects of VAC therapy on various wound types and demographics. It may also be interesting to investigate the role of adjunctive therapies like antibiotics or growth factors in combination with VAC therapy. Longitudinal analysis of long-term outcomes of wound healing and quality of life would give additional valuable insights into the persistence of the VAC therapy's impact.

## Conclusions

The study investigated outcomes in patients with complex wounds following reconstructive surgery that involved VAC therapy. There was a significant percentage of patients who improved, but by the end of the study, only 23.3% of the wounds healed. The VAC therapy, along with adjunctive interventions, such as flap surgery and skin grafts, appeared to be effective on various levels depending on the level of wound severity and comorbidity. Even though it is challenging for treatment, especially in cases of diabetic and cardiovascular patients, it offers a bright future perspective regarding the effectiveness of VAC therapy in promoting wound healing and management of complications.

## References

[1] Älgå A, Haweizy R, Bashaireh K, Wong S, Lundgren KC, von Schreeb J, Malmstedt J (2020). Negative pressure wound therapy versus standard treatment in patients with acute conflict-related extremity wounds: a pragmatic, multisite, randomised controlled trial. Lancet Glob Health.

[2] Armstrong DG, Lavery LA, Diabetic Foot Study Consortium (2005). Negative pressure wound therapy after partial diabetic foot amputation: a multicentre, randomised controlled trial. Lancet.

[3] Armstrong DG, Lavery LA, Boulton AJ (2007). Negative pressure wound therapy via vacuum-assisted closure following partial foot amputation: what is the role of wound chronicity?. Int Wound J.

[4] Arundel C, Buckley H, Clarke E (2016). Negative pressure wound therapy versus usual care for Surgical Wounds Healing by Secondary Intention (SWHSI trial): study protocol for a randomised controlled pilot trial. Trials.

[5] Burusapat C, Sringkarawat S (2021). Efficacy of negative-pressure wound therapy with tetrachlorodecaoxygen-anion complex instillation compared with standard negative-pressure wound therapy for accelerated wound healing: a prospective, randomized, controlled trial. Plast Reconstr Surg.

[6] Costa ML, Achten J, Knight R, Campolier M, Massa MS (2024). Five-year outcomes for patients sustaining severe fractures of the lower limb from the Wound Healing in Surgery for Trauma (WHIST) trial. Bone Joint J.

[7] Davis KE, La Fontaine J, Farrar D, Oz OK, Crisologo PA, Berriman S, Lavery LA (2020). Randomized clinical study to compare negative pressure wound therapy with simultaneous saline irrigation and traditional negative pressure wound therapy for complex foot infections. Wound Repair Regen.

[8] Engelhardt M, Rashad NA, Willy C, Müller C, Bauer C, Debus S, Beck T (2018). Closed-incision negative pressure therapy to reduce groin wound infections in vascular surgery: a randomised controlled trial. Int Wound J.

[9] Fan W, Hou F, Xi K, Hao C, Lu X, Zhao B (2021). A filled chocolates technique to seal negative-pressure wound therapy around external fixation devices: a randomized controlled trial. J Orthop Surg Res.

[10] Kamamoto F, Lima AM, de Rezende MR (2017). A new low-cost negative-pressure wound therapy versus a commercially available therapy device widely used to treat complex traumatic injuries: a prospective, randomized, non-inferiority trial. Clinics (Sao Paolo).

[11] Kutovoĭ AB, Kosul'nikov SO, Tarnopol'skiĭ SA, Karpenko SI, Kravchenko KV (2011). Treatment of purulent wounds using vacuum-therapy (Article in Russian). Klin Khir.

[12] Moffatt CJ, Murray S, Aubeeluck A, Quere I (2019). Communication with patients using negative wound pressure therapy and their adherence to treatment. J Wound Care.

[13] Ousey KJ, Milne J, Cook L, Stephenson J, Gillibrand W (2014). A pilot study exploring quality of life experienced by patients undergoing negative-pressure wound therapy as part of their wound care treatment compared to patients receiving standard wound care. Int Wound J.

[14] Pérez-Acevedo G, Torra-Bou JE, Peiró-García A, Vilalta-Vidal I, Urrea-Ayala M, Bosch-Alcaraz A, Blanco-Blanco J (2024). Incisional negative pressure wound therapy for the prevention of surgical site complications in Paediatric patients with non-idiopathic scoliosis: a randomized clinical trial. Int Wound J.

[15] Perez D, Bramkamp M, Exe C, von Ruden C, Ziegler A (2010). Modern wound care for the poor: a randomized clinical trial comparing the vacuum system with conventional saline-soaked gauze dressings. Am J Surg.

[16] Seidel D, Lefering R (2022). NPWT resource use compared with conventional wound treatment in subcutaneous abdominal wounds with healing impairment after surgery: SAWHI randomized clinical trial results. Ann Surg.

[17] Sepúlveda G, Espíndola M, Maureira M (2009). Negative-pressure wound therapy versus standard wound dressing in the treatment of diabetic foot amputation. a randomised controlled trial (Article in Spanish). Cir Esp.

[18] Sheng X, Hu L, Li T (2024). Clinical efficacy and mechanism of the combination of autologous platelet-rich gel and recombinant human acidic fibroblast growth factor in the management of refractory diabetic foot. Front Endocrinol (Lausanne).

[19] Stannard JP, Robinson JT, Anderson ER, McGwin G Jr, Volgas DA, Alonso JE (2006). Negative pressure wound therapy to treat hematomas and surgical incisions following high-energy trauma. J Trauma.

[20] Tapking C, Endlein J, Warszawski J (2024). Negative pressure wound therapy in burns: a prospective, randomized-controlled trial. Burns.

[21] Visser R, Milbrandt K, Lum Min S, Wiseman N, Hancock BJ, Morris M, Keijzer R (2017). Applying vacuum to accomplish reduced wound infections in laparoscopic pediatric surgery. J Pediatr Surg.

[22] Zhou M, Qi B, Yu A, Pan Z, Zhu S, Deng K, Tao S (2013). Vacuum assisted closure therapy for treatment of complex wounds in replanted extremities. Microsurgery.

